# The Synergistic Behavior of Antioxidant Phenolic Compounds Obtained from Winemaking Waste’s Valorization, Increased the Efficacy of a Sunscreen System

**DOI:** 10.3390/antiox8110530

**Published:** 2019-11-07

**Authors:** Alexandra Hubner, Flávia Sobreira, Alberto Vetore Neto, Claudinéia Aparecida Sales de Oliveira Pinto, Michelli Ferrera Dario, Ingrit Elida Collantes Díaz, Felipe Rebello Lourenço, Catarina Rosado, André Rolim Baby, Elfriede Marianne Bacchi

**Affiliations:** 1 School of Pharmaceutical Sciences, University of São Paulo, São Paulo/SP 05508-900, Brazil; alexandra.hubner@hotmail.com (A.H.); flaviacs@usp.br (F.S.); alberto.vetore.neto@usp.br (A.V.N.); clausal@usp.br (C.A.S.d.O.P.); mfdario@gmail.com (M.F.D.); feliperl@usp.br (F.R.L.); elfriede@usp.br (E.M.B.); 2 Facultad de Ingeniería Química y Têxtil/Facultad de Ciencias, Universidad Nacional de Ingeniería, Lima 15333, Peru; ingrit@iq.usp.br; 3 CBIOS – Universidade Lusófona’s Research Center for Biosciences and Health Technologies, Lisbo 1749-024, Portugal; p1657@ulusofona.pt

**Keywords:** *Vitis vinifera* L., grape pomace, phenolics, antioxidant activity, industrial waste valorization, sunscreens, sun protection factor

## Abstract

Grape pomace retains polyphenols in the peels and in the seeds after winemaking, which is indicative of the high valorization potential of this industrial waste. There is strong evidence that phenolics are robust antioxidants and confer photoprotection; thus, it is rational to apply these active compounds from winemaking waste to sunscreens, in order to increase UV protection. Despite the importance of this class of cosmetics to public health, more efficacious strategies are still needed to overcome the problems caused by the photoinstability of some UV filters. The hydroethanolic extract of *Vitis vinifera* L. grapes was obtained by percolation and then lyophilized. Six formulations were developed: Type I—cosmetic base and UV filters; Type II—cosmetic base and extract; and Type III—cosmetic base, extract and UV filters. Each formulation was prepared in the pHs 5 and 7. The antioxidant activities of the samples were measured by DPPH• and expressed in Trolox^®^ equivalents (TE), and their photostability and in vitro sun protection factor (SPF) were analyzed by diffuse reflectance spectrophotometry. The anti-radical efficiencies observed in the formulations with grape extract were: (II) 590.12 ± 0.01 μmol TE g^−1^ at pH 5 and 424.51 ± 0.32 μmol TE g^−1^ at pH 7; (III) 550.88 ± 0.00 μmol TE g^−1^ at pH 5 and 429.66 ± 0.10 μmol TE g^−1,^ at pH 7, demonstrating that the UV filters, butylmethoxydibenzoyl methane, ethylhexyl methoxycinnamate and ethylhexyl dimethyl 4-aminobenzoic acid had no influence on this effect. The photoprotective efficacy and the photostability of formulation III containing the extract and UV filters at pH 5 suggested that a synergism between the active molecules provided an 81% increase in SPF. Additionally, this was the only sample that maintained a broad spectrum of protection after irradiation. These results confirmed that the grape pomace extract has multifunctional potential for cosmetic use, mainly in sunscreens, granting them superior performance.

## 1. Introduction

Grapes were introduced in southern region of Brazil in the 19th century with the arrival of Italian immigrants to Serra Gaúcha, who had the tradition of producing wine for their own consumption [1]. *Vitis vinifera* L. is one of the most frequently-cultivated grape species, and it is economically important for manufacturing food, wine and other beverages. Recently, other uses began to be explored due to its chemical and widely-applicable characteristics [2], combined with high biocompatibility. Several cosmetics containing grape extracts have been successfully marketed.

Incomplete extraction of compounds such as polyphenols during winemaking, leads to about 70% of the initial active substances remaining in grape pomace waste [3], 20–30% in peels and 60–70% in seeds [4,5]. Considering the bioactive potentials of these polyphenols, especially flavonoids, the valorization of this type of industrial waste provides a natural raw material of relatively low cost and wide availability [6]. Grapes are rich in antioxidants with anti-inflammatory, antiallergenic, anticarcinogenic, cardioprotective, antithrombotic and hair-stimulating activities [7]. The antioxidant activity found in grape extracts has been attributed to the presence of phenolic compounds [8], including the large class of flavonoids with low molecular weights, that shield the plant from photodamage. There is evidence that these antioxidant molecules can also provide a photoprotective effect, which provides their application in sunscreens [9].

The contribution of this class of cosmetics to public health has risen in the last decade, due to an increased awareness of the importance of preventive measures in the fight against photodamage. Despite recent advances in sunscreen formulations, more efficacious strategies are still needed to overcome the problems caused by the photoinstability of some UV filters [10]. 

The purpose of our study was to further explore the antioxidant and photoprotective capacity of grape-pomace extract by assessing its application in a sunscreen. The phenolic compounds in the crude extract were identified by high performance liquid chromatography and electrospray tandem mass spectrometry (HPLC-ESI-MS/MS). The in vitro sun-protection factor (SPF) and the photostability of a formulation containing grape-pomace extract was measured using a diffuse reflectance spectrophotometer with an integration sphere. Additionally, we determined the total phenol content, expressed in gallic acid equivalents (GAE); the flavonoid content by reactions with aluminum trichloride, expressed as quercetin equivalents (QE); and the antioxidant activity by DPPH• inhibition assay, expressed in Trolox^®^ equivalents (TE). 

## 2. Materials and Methods

### 2.1. Chemicals and Reagents

Butylmethoxydibenzoylmethane and vinylpyrrolidone copolymer were purchased from Pharma Special (São Paulo, Brazil). Ethylhexyl methoxycinnamate and ethylhexyl dimethyl 4-aminobenzoic acid (ethylhexyl dimethyl PABA) were obtained from Fragon (São Paulo, Brazil). Phenoxyethanol/parabens and anhydrous citric acid were obtained from Vital Especialidades (São Paulo, Brazil). 2,2-Diphenyl-1-picrylhydrazyl (DPPH•), Folin-Ciocalteu reagent, aluminum chloride hexahydrate (AlCl_3_, 99%), gallic acid, (±)-6-hydroxy-2,5,7,8-tetramethylchromane-2-carboxylic acid-Trolox^®^, 2-(3,4-dihydroxyphenyl)-3,5,7-trihydroxy-4H-1-benzopyran-4-one and 3,3′,4′,5,6-pentahydroxyflavone (quercetin ≥ 95%) were from Sigma-Aldrich (São Paulo, Brazil). Sodium hydroxide, sodium carbonate, acetic acid and methanol were purchased from Synth (São Paulo, Brazil). Ethanol 96% (*v/v*) was purchased from LS Chemicals (São Paulo, Brazil), and distilled.

### 2.2. Plant Material

Plant material consisted of grape pomace from Cabernet Sauvignon (*V. vinifera* L.), mainly of skins and seeds and with a minority of stalks. The fruits were harvested in March 2014 and collected by a winegrower in the Valley of the Vineyards (29.2269° S, 51.1131° W) in the city of Caxias do Sul, RS, Brazil. The harvest was properly transported to the Beraldo Di Cale winery in Jundiaí, SP, Brazil. After maceration and alcoholic fermentation of mash for winemaking, the solid waste (fermented grape pomace) was separated from the liquid part for this research. The pomace arrived damp and it was dried in an oven with air circulation (Fabbe^®^, São Paulo, Brazil) at 40 °C for 5 days. The exsicata was deposited in the Herbarium of the Institute of Biosciences at USP/SP, with Hübner identification number A1.

A dried plant sample weighing 3.2 kg was ground in a knife and hammer mill, filtered through the pores in the 1.0 mm sieve and later processed with ball mill. The hydroethanolic-lyophilized extract was prepared with dry material, using a method adapted from the Brazilian Pharmacopoeia [11]. In brief, the powdered pomace remained in contact with the 70% hydroethanolic solution (*v/v*) by maceration for 18 h, and was subsequently held for percolation. The percolate was concentrated at a reduced pressure in an ascending film evaporator and then homogenized. The remaining aqueous solution was dried in a Liotop^®^ K202 lyophilizer (Liobras Industry Co., São Paulo, Brazil).

### 2.3. Analysis of Grape Pomace’s Dry Extract

#### 2.3.1. Quantification of Phenolics and Flavonoids

Phenolic content was determined by Folin–Ciocalteu (FC) reagent with gallic acid as the standard [12]. Samples were prepared in 10% ethanol at a concentration of 400 µg mL^−1^ for the extract and standard dilutions of gallic acid (85 to 25 µg mL^−1^). A 20 μL aliquot of sample or standard was added to each well of a 96-well plate, and then 30 µL of distilled water, 100 µL of 700 mM sodium carbonate and 50 mL of FC were added. 

Flavonoid content was determined using aluminum trichloride reagent (AlCl_3_) and quercetin for the standard [13,14]. The solutions were prepared in 60% (*v/v*) ethanol at a concentration of 17.5 mg mL^−1^ with pomace extract and standard dilutions of quercetin (50 to 200 µg mL^−1^). An aliquot of 20 µL of extract or standard in 60% (*v/v*) hydroethanolic solution was added to 20 µL of 2% AlCl_3_ in each well of a 96-well plate, followed by 210 µL of 60% (*v/v*) ethanolic solution. The absorbance of each sample was measured in triplicate with a Synergy HT Multi-Mode Microplate Reader (BioTek Industry Co., Winooski, VT, USA) at 765 (phenolic content) and 427 nm (flavonoid content), after a two-hour and thirty-min incubation protected from light. 

#### 2.3.2. In Vitro Antioxidant Activity

The antioxidant activity of the extract was determined by using the free radical 2,2-diphenyl-1-picryl-hydrazyl (DPPH•) in methanol and Trolox^®^, as a standard [15]. Crude extract was solubilized in methanol at concentrations of 1.0 mg mL^−1^ and subjected to ultrasound for 20 min. Then, solutions were adjusted to pH values of 5 and 7. Sample aliquots (0.1 mL) were added to 3.9 mL of a methanol solution containing 70 µM DPPH• and homogenized, and the mixtures were left standing at room temperature (22.0 ± 2.0 °C) protected from light for 30 min. Afterwards, solutions were evaluated by Thermo Scientific Evolution^®^ 600 spectrophotometer (Thermo Fisher Scientific Industry Co., Waltham, MA, USA) at a wavelength of 517 nm in quartz cuvettes with a 1.0 cm path length, in triplicate. The antioxidant activity was compared to the linear regression analyses (*R*^2^ = 0.9941) of standard Trolox solution (250 to 25 µg mL^−1^) and expressed as the mean ± standard deviation (*n* = 3) in Trolox equivalents (mg QE g^−1^ extract). 

#### 2.3.3. HPLC and ESI–MS

Phenolic analysis of total grape pomace (skins, seeds and stalks) was performed with a methanol solution with 10 mg mL^−1^ crude extract that was solubilized with ultrasound, filtered through a 0.45 µm nylon filter and injected into the loop (20 µL) at a flow rate of 0.7 mL min^−1^ at ambient temperature [16]. The polyphenols were separated in a C18 reversed-phase column, Shim-pack VP-ODS (250 × 4.6 mm; 5 µm particle size) with a system controller CBM-20A (Shimadzu Co., Kioto, Japan) connected to binary pumps LC-20AD (Shimadzu^®^) that had a UV/VIS SPD-20A detector (Shimadzu^®^), CTO-20A (Shimadzu^®^) and self-injector SIL 20AC (Shimadzu^®^). The solvent system used to elute the compounds was: A—0.1% acetic acid water solution, and B—methanol. The following gradient conditions were used: 0–10 min (95% A/5% B); 10–60 min (50% A/50% B); 60–80 min (30% A/70% B); and 80–90 min (95% A/5% B). A chromatographic profile of the extracts was obtained at a wavelength of 254 nm. The identification of the compounds in grape pomace was performed in an AmaZon ETD (Bruker Co., Billerica, MA, USA) instrument with positive ion mode mass spectrometry using 27 psi and a capillary voltage of 4500 V.

### 2.4. Formulations

A total of 6 cosmetic preparations with and without dry extract of red grape pomace were prepared based on a hydrophilic copolymer of ammonium acryloyldimethyltaurate vinylpyrrolidone (cosmetic base). The UV filters—butylmethoxydibenzoyl methane, a UVA filter, ethylhexyl methoxycinnamate, a UVB filter and ethylhexyl dimethyl PABA, a UVB filter—were used. These emulsions are described qualitatively and quantitatively (% *w/w*) in Table 1. Each formulation (Types I, II and III) was adjusted to pH values of 5 and 7, and the UV filter concentration was in accordance with international legislation and guidelines [17,18,19]. All samples were prepared in triplicate.

#### 2.4.1. In Vitro Antioxidant Activity of the Formulations

The antioxidant activity of the formulations (F1–F6) was examined according to the method described in item 2.3.2 [15,20,21,22]. Sample aliquots of formulations II and III (1 g) containing 10% crude pomace extract were solubilized in methanol at concentrations of 1 mg mL^−1^ and sonicated for 20 min. Then, samples were centrifuged at 2500 rpm for 20 min, and pH adjusted to 5 and 7. The supernatants were used in the spectrophotometric analyses. A 100 µL aliquot of the diluted formulation was added to 3.9 mL of a 70 μM DPPH• methanolic solution and homogenized. As described earlier, antioxidant activity was expressed in Trolox equivalents (mg TE g^−1^ extract) as the mean ± standard deviation (*n* = 3). 

#### 2.4.2. Photoprotection Efficacy and Photostability

The sun protection factor (SPF) of F1 to F6 was determined in vitro with a diffuse reflectance spectrophotometer with an integration sphere (Labsphere Inc., North Sutton, NH, USA) [22,23,24]. Samples were weighed on an analytical balance (Shimadzu^®^ AUY 220) and uniformly applied over polymethylmethacrylate (PMMA) (HelioScreen^®^ Helioplate HD 6, North Sutton, NH, USA) plates at 0.75 mg cm^–2^, kept at room temperature (22.0 ± 2.0 °C) and protected from the light for 15 min. Subsequently, the absorbance of the photoprotective formulations was determined using the wavelength range of 290–400 nm in triplicate, with seven different points of readings per plate of each sample. The average spectral absorbance values were used to calculate the SPF, critical wavelength and UVB/UVA rate of the formulations [25,26,27,28] by the UV2000^®^ software (North Sutton, NH, USA).

A photostability test was performed to predict possible degradations due to the artificial radiation emitted by the photostability chamber. A total of 0.0187 g of each sample was weighed and applied over PMMA plates, in triplicate. Readings in Labsphere^®^ UV-2000S Transmittance Analyzer, as described, were at 0, 1 and 2 h after sun exposure simulated by Suntest CPS + (Atlas Co., Linsengericht, Germany) which provided a radiation dose of 500 W m^−2^ with a temperature of 35 °C [26,27,28].

### 2.5. Statistical Analysis

Results were evaluated by one-way analysis of variance (ANOVA) followed by Tukey’s test. Additionally, two-way ANOVA followed by Sidak’s post hoc test and a Student’s *t*-test with a confidence interval at 95% (*p* < 0.05) were performed using Minitab version 17 and GraphPad Prism 6.0 statistical software (San Diego, CA, USA).

## 3. Results and Discussion

### 3.1. Analysis of Grape Pomace’s Dry Extract

#### 3.1.1. Quantification of Phenolics and Flavonoids

Grapes are sources of phenolic compounds that are extracted from seeds and skins during winemaking. However, this extraction into the wine is incomplete and grape pomace retains a high amount of such substances [29]. According to the literature, the wide variation in the phenolic content of *V. vinifera* grapes can be attributed to cultivation constraints, such as (a) viticulture conditions, genetic grapevine, maturation stage, edaphoclimatic factors and exposure to fungi; (b) oenology parameters, time and storage temperature of postharvest samples, type and length of maceration, pressing and fermentation; and (c) winemaking extraction process, cultivar, variety, sample type, drying, solvent system, volume, time, milling, temperature, pH value, metal ions, light and oxygen exposure and extraction technologies. It is highly likely that these factors change the chemical and physical characteristics of the coproduct, and thus, their extensive biological activities [26,27,28,29,30,31,32,33,34]. 

The most common phenolic compounds derived from grapes are flavonoids, such as anthocyanins, flavanols and flavonols [33]. Innumerable biological and pharmacological activities of flavonoids against cellular and tissue injury have been described [34], such as anti-inflammatory, antioxidant [2,35], anti-tumor, anti-allergic, anti-thrombotic, anti-diabetic and anti-atherosclerotic [36]. Due to a favorable combination of bioactivity and safety, flavonoids from different plants have been used in cosmetic products to combat skin aging, pollution and UV damage [33,36]. In the present study, the total phenolic content from hydroethanolic extract was 3.01 ± 0.14 mg QE g^−1^ and the total flavonoid content was 141.11 ± 3.38 mg GAE g^−1^.

#### 3.1.2. In Vitro Antioxidant Activity

**Figure 1:**
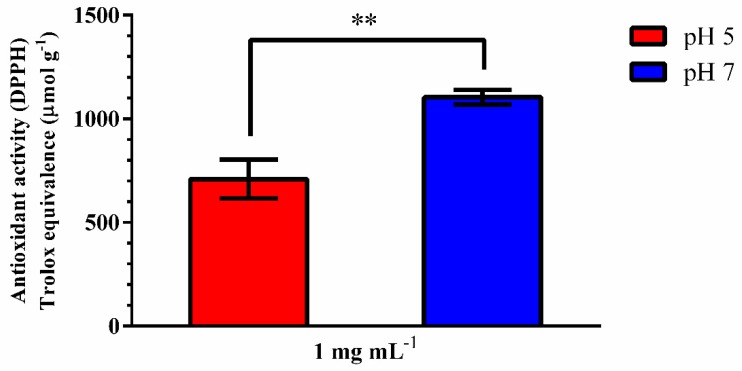
Antioxidant activity of 1.0 mg mL^−1^ grape pomace extract (*Vitis vinifera*) at pH values 5 and 7 (*n* = 3). ** = *p* < 0.001.

Interestingly, the antioxidant activity of the hydroethanolic extract of grape pomace changed significantly at different pH values, being 707.00 ± 0.03 and 1098.00 ± 0.01 μmol TE g^−1^ at pHs 5 and 7, respectively (Figure 1). The antioxidant activity of red grapes has been associated with polyphenolic content [37,38]. Grape pomace’s (seeds, skin and peduncle) hydroethanolic extracts (water/ethanol 60%) were evaluated after crushing, pre-fermentation and after the first fermentation. The antioxidant activity of the winery pomace decreased by approximately 36%, in comparison to the grape pomace, which was attributed to a change in the phenolic composition due to the extraction of these into the wine [38]. In our study, the 70% hydroethanolic extracts had an almost 10-fold superior antioxidant activity compared to the results from Salazar and collaborators [38]. The 70% alcoholic solution may have facilitated the release of less soluble and more stable substances, improving the antioxidant effect. [3]. In another research, grape pomace extracts of white and red *V. vinifera* prepared with water/acetone exhibited a range of free radical scavenging activity from 3.54 ± 0.06 to 28.20 ± 0.02 μmol TE g^−1^. Such a reduced antioxidant activity may have been due to the extraction with acetone, a less polar solvent. The red grape pomaces had the highest DPPH• scavenging capacity due to differences in the polyphenolic content [39]. Literature indicates that the red grape varieties of the Mediterranean (Grenache, Syrah, Carignan, Mourvèdre and Alicante) have higher antioxidant activity; however, it should be noted that there were contrasts between the results for seeds and grape skins [3]. 

#### 3.1.3. HPLC and ESI-MS/MS

**Figure 2:**
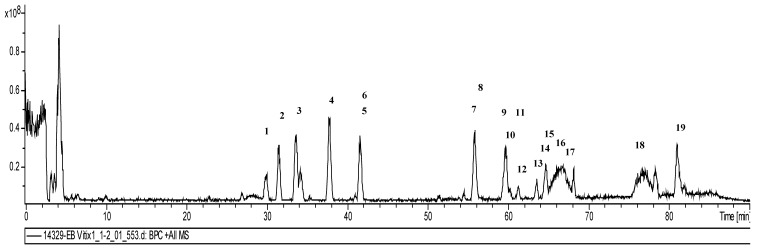
Base peak chromatogram (BPC) of the crude *Vitis vinifera* L. extract in positive mode with procyanidins (dimer and trimer) and the identified flavonoids.

The phenolic compounds identified by the mass spectrometric analysis of the crude extract included procyanidin dimers and trimers and thirteen compounds from three phenolic groups; namely, one dihydroflavonol, five flavonols and seven anthocyanins (Figure 2 and Table 2). In previous studies with the same and another kind of *V.vinifera* cultivars, the same compounds were identified. The peak at 29.9–41.5 min showed the dimers and trimer of B-type procyanidin. The fragment ions have well-known MS^1^ and MS^2^ fragmentation patterns caused by an established retro-Diels–Alder (RDA) mechanism, heterocyclic ring fission (HRF) and quinone methide (QM) cleavage [40,41,42].

### 3.2. Formulations

#### 3.2.1. In Vitro Antioxidant Activity of the Formulations

**Figure 3:**
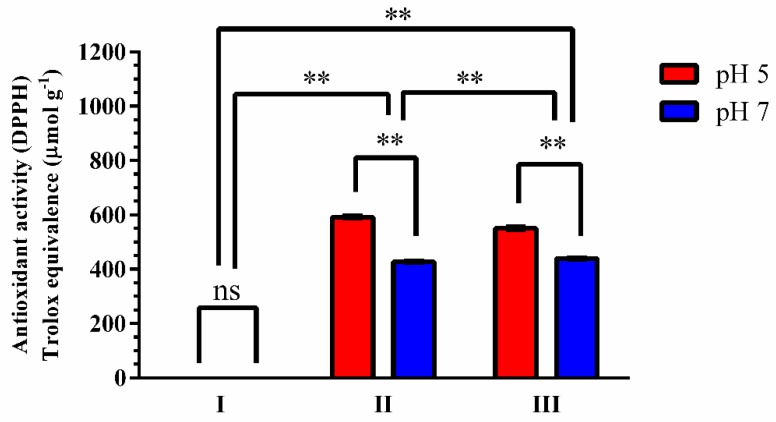
In vitro antioxidant activity of formulations containing grape pomace extract at pH values 5 and 7 (*n* = 3). I—base and UV filters; II—base and grape pomace; III—base, UV filters and grape pomace. The results were evaluated according to the statistical two-way ANOVA: pH (*p* < 0.001), concentration (*p* < 0.0001). Post hoc test Sidak. ** = significant (*p* < 0.001), and ns = not significant (*p* < 0.001).

In recent decades, personal care products formulated with plant extracts have gained significant interest due to the numerous health benefits [46]. As shown in Figure 3, differences (*p* > 0.001) were found between the formulations with and without grape pomace extract. However, no differences were seen in the antioxidant activities of the formulations with grape pomace’s extract with and without UV filters at the same pH value: formulation II—590.12 ± 0.01 μmol TE g^−1^ at pH 5 and 424.51 ± 0.32 μmol TE g^−1^ at pH 7; formulation III—550.88 ± 0.00 μmol TE g^−1^ at pH 5 and 429.66 ± 0.10 μmol TE g^−1^, at pH 7. It should be noted that, unlike the results obtained for the grape pomace extracts, the activity was lower at pH 7. 

#### 3.2.2. In Vitro Photoprotection Efficacy and Photostability

SPF can be measured in vitro using artificial substrates and spectrophotometric techniques, allowing the screening of the photoprotective efficiency of active compounds and sunscreen formulations against skin damage caused by UV radiation [47,48]. Additionally, these in vitro methods are efficient, economically convenient and ethical [49,50].

**Figure 4:**
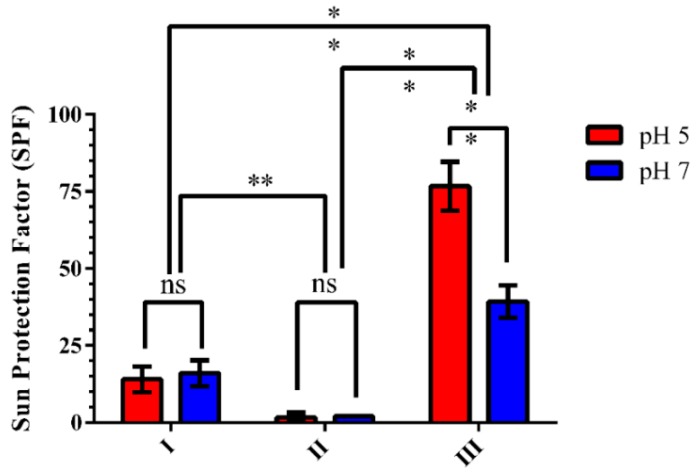
In vitro sun protection factor of formulations I, II and III containing grape pomace extract at two pH values 5 and 7 (*n* = 3). pH (*p* < 0.001), concentration (*p* < 0.001) and interaction (*p* < 0.001). Post hoc Sidak test. Formulation I—base and UV filters; II—base and grape pomace; III—base, UV filters and grape pomace. ** = significant (*p* < 0.001), * = significant (*p* < 0.01) and ns = not significant.

Results showed that formulation Type III, that combined filters and grape pomace extract, was more effective against UVA and UVB radiations than the other samples, reaching SPF values above 76 and the critical wavelength of 380.00 nm at pH 5. As expected, no SPF was achieved with the formulations without UV filters (Type II). The pH values of each formulation Type III sample influenced SPF (Table 3 and Figure 4). It is interesting to note that these results were in accordance with the in vitro antioxidant activity of the formulations described in the previous section. It is possible that such an increase was also related to an increased stability of polyphenolic compounds and changes in displacement of hyperchromic and bathochromic absorption bands at lower the pH [19,51].

Table 3 shows the SPF and critical wavelength values of the formulations containing grape pomace at different pH values and irradiation times. Not only did formulation Type III provide the highest SPF values, but it was also able to maintain a good protection after 2 h of irradiation (SPFs around 17 and 12 for pH 5 and 7, respectively, and critical wavelength around 376 nm for both). In contrast, formulation Type I containing only UV filters provided lower initial SPF values, and after 2 h was only just above the minimum labeled SPF of 6.00 and critical wavelength of 370.00 recommended by the main regulatory agencies [28,50]. According to the guidelines, a broad-spectrum sunscreen protecting against UVA and UVB radiation has to achieve SPF ≥ 15.00 and critical wavelength ≥ 370.00 nm [28,50]. It is well known that there are other factors that may interfere with the final SPF of a sunscreen formulation. Not only the concentration and combination of UV filters are critical, but also the emulsifiers, vehicles and solvents used, the viscosity of the vehicle, active-vehicle, active-skin and vehicle-skin interactions and others [52,53].

Formulating sunscreens with chemical UV filters is challenging due to their photoinstability. Butylmethoxydibenzoyl methane, which attenuates UVA radiation at 340–400 nm, is commonly used in photoprotective products, but can be degraded by approximately 50–90% after 60 min of UV exposure [54]. Thus, one of the formulation strategies to obtain a broad spectrum of UV protection and/or photostability in cosmetic products is the combination of organic filters or a blend of organic and inorganic filters. For example, the combination of ethylhexyl methoxycinnamate and ethylhexyl dimethyl PABA iswidely used in cosmetics against UVB radiation in the 290–340 nm range [54,55]. However, filter associations must be selected cautiously, since there are combinations of filters that can result in instability of the system and the formation of photodegradation byproducts. Another problem related to UV filters is that some sunscreens and UV stabilizers may migrate into sea and groundwater and cause environmental damage [56,57]. For these reasons, there is a current trend in the application of natural products to photoprotective systems to reduce their adverse effects on consumers and the environment.

Flavonoids, alone, do not sufficiently attenuate or suppress the photochemical reactions catalyzed by UV radiation, but the synergism between flavonoids and UV filters may reduce the formation of deleterious degradation photoproducts; thus, boosting the photoprotection provided [58,59]. For example, in a study using passion fruit seed extract (*P. edulis*) from juice processing, the authors reported a synergistic effect of the flavonoids rich extract with inorganic pigments, consequently increasing the SPF [49].

In our work, the SPF values obtained in the formulation Type III containing extract and filters showed the highest protection factors pre-irradiation (T0). After 2 h of exposure to artificial UV, samples suffered some photodegradation; however, these values were two to three-fold higher than those observed in formulation I, which only contained UV filters. In a similar study, a stability improvement was detected for the polyphenolics from the skins of the red grape Cabernet Sauvignon (*V. vinifera* L.) at an acidic pH [60], while another preliminary study observed that products with poor photostability may have overestimated in vitro SPF values [61]. In the work of Martincigh et al., ethylhexyl methoxycinnamate, benzophenone-3 and tert-butyl-methoxydibenzoylmethane in the presence of grape seed extract improved the photostability of a methanol solution by 16.90% after 4 h of UV irradiation. According to a previous study, grape seed extract produced photoproducts through a radical deprotonation reaction, and these synergistically interacted with chemical filters, improving UV protection [53].

## 4. Conclusions

A high antioxidant activity for formulations Type II or III at both pH 5 and 7 values was observed, the activity being attributable to the phenolic substances identified in *V. vinifera* L. extract, including procyanidine, dihydroflavoninol, flavonols and anthocyanins dimers and trimers, since the antioxidant response was not significant in the formulation without extract (I). The SPF values of sample I, before irradiation, at pH 5 and 7, were about 81% and 59% lower, respectively, than sample III. The comparison between the results obtained with formulations II and III suggested a synergistic behavior with the extract and the sunscreen system. The photoprotective response of formulation III after 2 h irradiation at pH 5 was 2.5 times higher than that of the formulation I in same pH; in addition, it was the only one that maintained good properties post-artificial UV irradiation. Therefore, our results are promising and indicate that the antioxidants obtained from the winemaking residue can be used to increase the efficiency of sunscreens without affecting ecosystems, and while using sustainable hydroethanolic extraction, and enabling the economic and social development of renewable sources.

## Figures and Tables

**Table 1:** Qualitative and quantitative composition (% *w/w*) of the sunscreens with and without grape pomace extract (F1 to F6) at two pH values.

Ingredients (INCI ^a^)	Concentration (% *w/w*)					
	Type I		Type II		Type III	
	F1 – pH 5	F2 – pH 7	F3 – pH 5	F4 – pH 7	F5 – pH 5	F6 – pH 7
**Oil phase**						
Ethylhexyl methoxycinnamate	10.0	10.0	-	-	10.0	10.0
Ethylhexyl dimethyl PABA	10.0	10.0	-	-	10.0	10.0
Butyl methoxy dibenzoyl methane	5.0	5.0	-	-	5.0	5.0
Mixture of phenoxyethanol and paraben esters *	0.1	0.1	0.1	0.1	0.1	0.1
**Aqueous phase**						
Ammonium acryloyldimethyltaurate vinylpyrrolidone	2.0	2.0	2.0	2.0	2.0	2.0
Grape pomace extract of *V. vinifera* L.	-	-	10.0	10.0	10.0	10.0
Purified water	72.9	72.9	87.9	87.9	62.9	62.9

^a^ INCI: International Nomenclature of Cosmetic Ingredients. * methylparaben, ethylparaben, propylparaben, butylparaben, and isobutylparaben. Formulations (I, II and III) were prepared with pH values of either 5 and 7 with citric acid or sodium hydroxide (sufficient quantities).

**Table 2:** ESI-MS/MS of the phenolic compounds identified in the crude *Vitis vinifera* L. extract.

Peak	MW (Da)	R_T_ (min)	[M+H]^+^ (m/z)	MS/MS (m/z)	Major Fragment Ion (m/z)	Formula	Peak Identity	References
**1**	578	29.9	579.26	561.34, 453.31, 427.23, 409.24, 291.10, 247.05	[M+H−H_2_O]^+^, [M+H−HRF_C_]^+^, [M+H−RDA]^+^, [M+H−RDA−H_2_O]^+^, [M+H−QM]^+^	C_30_H_26_O_12_	B-type procyanidin dimers	[40,41,42]
**2**	578	31.6	579.26	543.33, 409.25, 291.10, 247.05, 200.88				
**3**	578	33.7	579.25	561.19, 409.17, 291.11, 246,98				
**4**	578	37.9	579.28	561.35, 453.29, 427.22, 409.23, 291.14				
**5**	578	41.5	579.30	561.33, 453.31, 409.23, 291.11, 247.02, 164.98				
**6**	866		867.38	697.34, 579.33, 409.25, 289.15	[M+H−RDA−H_2_O]^+^, [M+H−QM_C_]^+^, [M+H−QM−RDA−H_2_O]^+^, [M+H−QM_CD_]^+^	C_45_H_38_O_18_	Trimer procyanidins	
**7**	450	55.8	451	415.19 305.06	[M+H−2H_2_O]^+^ [M+H−Rham]^+^	C_21_H_22_O_11_	Di-hydroxyquercetin-*O*-rhamnose	[43,44]
**8**	478	55.9	479.20	303.10	[M+H−Gluc]^+^	C_21_H_18_O_13_	Quercetin-*O*-glucuronide	[40,45]
**9**	610	59.3	611.28	465.25 303.10	[M+H−Rham]^+^ [M+H-Rham−Glc]^+^	C_27_H_30_O_16_	Rutin	[45]
**10**	464	59.6	465.21	303.07	[M+H−Glc]^+^	C_21_H_18_O_13_	Quercetin-3-*O*-glucoside	[40,45]
**11**	560	59.7	561.14	399.21	[M+H−Glc]^+^	C_26_H_25_O_14_	Malvidin-3-*O*-glucoside pyruvate	[45]
**12**	462	61.2	463.25	287.09	[M+H−Gluc]^+^	C_21_H_18_O_12_	Kaempferol-3-*O*-glucuronide	[40,45]
**13**	302	63.4	303.15	303.08, 257.03	[M+H-H_2_O−CO]^+^	C_15_H_10_O_7_	Quercetin	[40,45]
**14**	478	64.4	479.22	317.15	[M+H−Glc]^+^	C_22_H_23_O_12_	Petunidin-3-*O*-glucoside	[43,44]
**15**	530	64.6	531.18	369.15	[M+H−Glc]^+^	C_25_H_23_O_13_	Peonidin-3-*O*-glucoside pyruvate	[45]
**16**	492	65.3	493.22	331.16	[M+H−Glc]^+^	C_23_H_25_O_12_	Malvidin-3-O-glucoside	[45]
**17**	678	67	679.33	661.69 585.31	[M+H−C_6_H_6_O]^+^ M+H−H_2_O]^+^	C_25_H_23_O_13_	Delphinidin-3-*O*-(6”-O-p-coumaryl) glucoside pyruvate	[45]
**18**	534	76.1	535.27	517.42 331.18	[M+H−2H_2_O]^+^ [M+H-acetylGlc]^+^	C_23_H_25_O_12_	Malvidin-3-(6”-*O*-acetylglucoside)	[45]
**19**	654	81.1	655.32	636.75 331.17	[M+H−H_2_O]^+^ M+H−caffeoylGlc]^+^	C_33_H_27_O_16_	Malvidin-3-(6”-*O*-caffeoylglucoside)	[45]

R_T_: retention time; MW: molecular weight; [M+H]^+^: molecular ion; MS^2^: fragment ions; RDA: retro-Diels–Alder; HRF: heterocyclic ring fission; and QM: quinone methide [42].

**Table 3:** In vitro SPF and critical wavelength (nm) values of sunscreens at different pH values and irradiation times.

Formulations		pH	SPF *			Critical λ **		
			T 0	T 1	T 2	T 0	T 1	T 2
**I**	F1	5.0	14.00 ± 1.70 ^H I^	7.67 ± 1.53 ^J K L M N^	6.67 ± 1.53 ^L M N O^	381.67± 0.60 ^A B^	380.33 ± 0.58 ^A B^	380.00 ± 1.00 ^A B^
	F2	7.6	16.00 ± 1.70 ^G H I^	7.67 ± 0.58 ^J K L M N^	6.00 ± 0.00 ^L M NO^	381.33± 0.60 ^A B^	379.67 ± 0.58 ^A B^	379.33 ± 0.58 ^A B^
**II**	F3	5.2	1.67 ± 0.58 ^O^	1.67 ± 0.58 ^O^	1.67 ± 0.58 ^O^	360.33± 1.50 ^D^	364.67 ± 2.08 ^C D^	366.00 ± 1.73 ^C D^
	F4	7.0	2.00 ± 0.00 ^O^	2.00 ± 0.00 ^O^	2.00 ± 0.0 ^O^	365.33± 2.10 ^C D^	368.67 ± 2.08 ^C^	369.00 ± 2.65 ^C^
**III**	F5	5.4	76.67 ± 3.21 ^B^	26.33 ± 1.53 ^E^	17.33 ± 0.58 ^F G H^	380.00± 0.00 ^A B^	377.67 ± 0.58 ^A B^	376.67 ± 0.58 ^A B^
	F6	7.2	39.33 ± 2.08 ^D^	16.67 ± 1.15 ^G H^	12.33 ± 0.58 ^H I J^	380.33± 0.60 ^AB^	377.67 ± 0.58 ^AB^	376.00 ± 1.00 ^B^

* Estimated sun protection factor; ** critical wavelength (nm); T – irradiation time (hours); I—cosmetic base + filters UVA and UVB; II—cosmetic base + grape pomace; and III—cosmetic base + UV filters + grape pomace. Different letters (A–O) represent statistically significant differences among groups. The results were expressed as the mean ± standard deviation (*n* = 3). Statistical differences were determined using one-way ANOVA followed by Tukey’s test for comparisons between groups (level of significance of 0.05).
